# The Economics of a Successful Raccoon Rabies Elimination Program on Long Island, New York

**DOI:** 10.1371/journal.pntd.0005062

**Published:** 2016-12-09

**Authors:** Julie L. Elser, Laura L. Bigler, Aaron M. Anderson, Joanne L. Maki, Donald H. Lein, Stephanie A. Shwiff

**Affiliations:** 1 USDA/APHIS/WS National Wildlife Research Center, Fort Collins, Colorado, United States of America; 2 New York State Veterinary Diagnostic Laboratory, Department of Population Medicine and Diagnostic Sciences, College of Veterinary Medicine, Cornell University, Ithaca, New York, United States of America; 3 Merial Inc., Athens, Georgia, United States of America; Centers for Disease Control and Prevention, UNITED STATES

## Abstract

Raccoon rabies is endemic in the eastern U.S.; however, an epizootic had not been confirmed on Long Island, New York until 2004. An oral rabies vaccination (ORV) program was initiated soon after the first rabies-positive raccoon was discovered, and continued until raccoon rabies was eliminated from the vaccination zone. The cost-effectiveness and economic impact of this rabies control program were unknown. A public health surveillance data set was evaluated following the ORV program on Long Island, and is used here as a case study in the health economics of rabies prevention and control efforts. A benefit-cost analysis was performed to determine the cost-effectiveness of the program, and a regional economic model was used to estimate the macroeconomic impacts of raccoon rabies elimination to New York State. The cost of the program, approximately $2.6 million, was recovered within eight years by reducing costs associated with post-exposure prophylaxis (PEP) and veterinary diagnostic testing of rabies suspect animals. By 2019, the State of New York is projected to benefit from the ORV program by almost $27 million. The benefit-cost ratio will reach 1.71 in 2019, meaning that for every dollar spent on the program $1.71 will be saved. Regional economic modeling estimated employment growth of over 100 jobs and a Gross Domestic Product (GDP) increase of $9.2 million through 2019. This analysis suggests that baiting to eliminate rabies in a geographically constrained area can provide positive economic returns.

## Author Summary

Raccoon rabies, a type of rabies found most frequently in raccoons, is common in the eastern U.S. Raccoon rabies is at times controlled through oral rabies vaccination (ORV) programs, in which baits containing rabies vaccine are distributed throughout the affected area to be consumed by wildlife, which then develop immunity limiting their ability to spread the disease in the population. A rabies outbreak was not detected in Nassau and Suffolk Counties on Long Island until 2004, when an ORV program was rapidly initiated in response to the discovery of a rabies-positive raccoon. As a result, raccoon rabies was eliminated in January 2009. Some questioned whether the benefits of the ORV program outweighed its costs. To find out, we performed a benefit-cost analysis of the program using avoided medical and animal testing costs to value the benefits. We also used a regional economic model to estimate the effect of the program on the economy of New York State. The results of both analyses indicated that the program had a positive economic impact, increasing employment and income in the region. The ORV program also benefited public health by reducing the need for medical treatment after possible rabies exposures. This study provides insight into the economic value of a program to eliminate rabies from an isolated area.

## Introduction

The raccoon variant of rabies virus (raccoon rabies) is endemic throughout most of the eastern seaboard of the U.S. In New York, raccoon rabies was first reported in 1990. The epizootic was undetected in Long Island (Nassau County) until August 2004 [1], excepting one isolated case in 1991. This delay may have been due to the presence of geographic barriers (East River and New York City) at the west end of the island. Rabies surveillance in Brooklyn and Queens is managed by the New York City Department of Health and Mental Hygiene. Animals are routinely tested by the Public Health Laboratory in New York City if potential viral exposure involving people or domestic animals has been reported, as well as when the animals are found sick (e.g., menacing behavior, neurological signs) in the environment. Rabies surveillance in the remaining counties of New York State is performed by the county health departments, including those located in Nassau and Suffolk Counties. Specimens are shipped to the New York State Rabies Laboratory (Wadsworth Center) for analyses. In the absence of ORV, routine passive rabies surveillance is typically limited only to those animals that have placed humans or domestic animals at risk of rabies infection through reports of direct or indirect contacts. However, rabies surveillance in Nassau and Suffolk Counties was further enhanced subsequent to the first case that was reported in 2004, to also include the submission of rabies-suspect animals with no reported contacts with people or domestic animals.

The number of rabies-positive raccoons in Nassau and Suffolk Counties (Long Island, NY) increased from zero in 2003 to ten in 2004 and 35 in 2005. To combat the growing number of rabid raccoons, the New York State Department of Health initiated an immediate response strategy to control rabies when rabid raccoons were first detected in Nassau County during August 2004. Rabies control was not achieved, as was evidenced by the increased number of rabid animals (N = 35) that were reported in Nassau County in 2005, as well as viral advance into Suffolk County by March 2006 (Table 1). In September 2006, the management of the Long Island Oral Rabies Vaccine (ORV) Program was transferred to the N.Y.S. Veterinary Diagnostic Laboratory (NYSVDL) at Cornell University. From 2005–2010, raccoon populations were vaccinated by broad-scale ground or aerial distribution of ORV-filled baits containing RABORAL V-RG rabies vaccine throughout the affected area. NYSVDL adopted a minimum target bait density of 250 baits/km^2^ during September applications. Additional research activities performed from 2007 to 2009 in approximately 20% of the ORV zone also incorporated the evaluations of semi-annual (i.e., July and September) ORV campaigns and greater, cumulative bait densities ranging up to 1,000 baits/km^2^. The data generated from the intensive ORV strategies did not support continuation of the experimental methodologies on Long Island. However, 100% of the vaccine costs (i.e., routine and research) have been included in our cost analysis, thereby inflating ORV expenses and decreasing estimated benefits. Raccoon rabies was eliminated from the ORV zone in 2009, reducing its impacts to human and animal health and the economy of New York State.

**Table 1:** Numbers of terrestrial rabid animals confirmed in Queens, Kings, Nassau and Suffolk counties (1991–2016 (July)).

County	1991	1993	2001	2004 *	2005	2006	2007 **	2008	2009 ***	2010	2011	2012	2013	2014	2015	2016	TOTAL
**Kings**	0	0	0	0	0	0	0	0	0	**2**	0	**1**	**2**	**5**	0	0	**10**
**Queens**	0	**2**	**1**	0	**1**	**2**	**1**	**1**	**1**	**2**	0	0	0	0	0	**1**	**12**
**Nassau**	**1**	0	0	**10**	**35**	**18**	**4**	0	0	0	0	0	0	0	0	**1**	**69**
**Suffolk**	0	0	0	0	0	**5**	**12**	**1**	**1**	0	0	0	0	0	0	0	**19**

*Enhanced rabies surveillance (i.e., submission and analysis of all rabies-suspect animals, including those individuals with no reports of contact with people or domestic animals) in progress within Kings, Queens, Nassau and Suffolk Counties in 2004 and thereafter

** Last historical case in Nassau County—November 2007

*** Last historical case in Suffolk County—January 2009

Wildlife Rabies Control Interventions:

White: NYS Department of Health—Northern Nassau, Suffolk and Queens Counties

Light gray: NYS Veterinary Diagnostic Laboratory—Northern Nassau and Eastern Suffolk Counties

Dark gray: NYS Veterinary Diagnostic Laboratory—Kings, Queens and Southwestern Nassau Counties

In the U.S., interactions between people and companion animals are an important cause of human rabies exposure [2]. Companion animals can be a source of rabies exposure concern when a person handles their unvaccinated dog or cat or is bitten or scratched by it after it has been found near or in contact with a rabid raccoon. Given that the case-fatality rate of rabies is nearly 100% and the disease is completely preventable through timely post-exposure prophylaxis (PEP), many individuals who are at very low risk of developing the disease still seek PEP, regardless of the recommendation of health professionals [3]. Therefore, the direct economic impacts of rabies are associated predominantly with human PEP [4]. Potential rabies exposure also has indirect impacts including vaccination of livestock and companion animals, livestock mortality, time off work to receive treatment, and testing of domestic and wild animals that may be infected with the virus [4, 5]. In this study, indirect costs included public health and animal control costs, as well as non-PEP patient costs such as travel to receive treatment and lost wages due to missing work.

Macroeconomic impacts of endemic disease burdens such as rabies can be estimated by examining changes in different economic sectors that result from the direct and indirect impacts of the disease [6]. For example, reductions in consumer spending throughout the economy can result from income lost when seeking PEP treatment. As these impacts spread through the regional economy, they affect members of the community who were not directly impacted by the disease.

The purpose of this study is to quantify the costs associated with the Long Island ORV program and the benefits gained from the resulting decrease in human and animal rabies exposure. Additionally, a regional economic model is used to estimate the macroeconomic impacts of the program in New York State. This study provides an assessment of the economic impacts of rabies and an estimate of the potential benefits that could be realized if the prevalence of raccoon rabies was reduced or eliminated in other regions of the U.S.

## Methods

Benefit-cost analysis (BCA) is a tool frequently used by economists to evaluate the efficiency of projects and government programs. Valuation of the benefits of projects that prevent disease spread is generally based on estimation of the damages avoided. It is posited that the ORV program described here decreased the prevalence of (and ultimately eliminated) raccoon rabies. The benefits of this ORV program were calculated as the savings from reducing the number of PEP and animal testing (AT) necessary, and the associated costs borne by individuals as a result of human rabies exposure (e.g., expenditures on over-the-counter medications, lost work time and travel to receive treatment). These avoided costs make up the majority of benefits derived from rabies control programs [7–9], and accrue to the people of New York State in the form of reduced expenditures on healthcare and reduced spending by the state and counties on animal testing.

### Benefit-Cost Analysis

**Fig 1:**
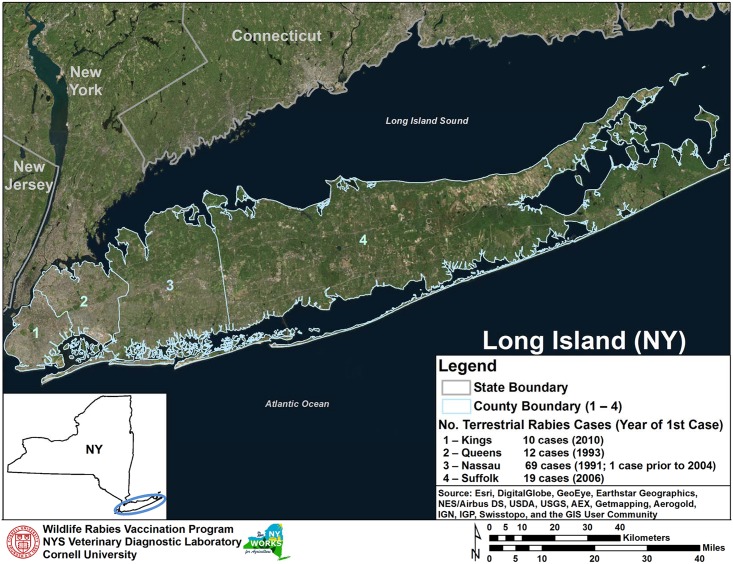
Habitat and first appearance of rabies by county on Long Island, NY.

**Fig 2:**
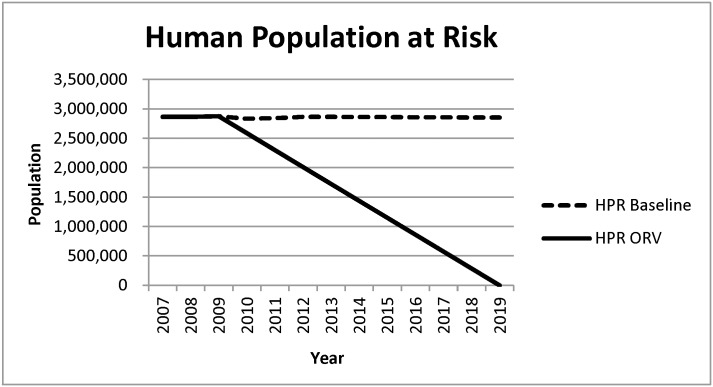
Human Population at Risk (*HPR*) with and without the ORV program.

The annual total benefits (*TB*) equal PEP and AT costs avoided due to raccoon rabies cases avoided. To determine cost savings it was necessary to estimate the extent to which raccoon rabies was likely to spread on Long Island. For this analysis, it was assumed that the disease would not severely impact Queens and Kings Counties due to the high level of urbanization, but would have spread east through Nassau and Suffolk Counties (Fig 1). As a result, with no effort to control raccoon rabies on Long Island, the total possible human population at risk (*HPR*_*baseline*_) comprises the populations of both Nassau and Suffolk Counties (Fig 2). Rabies prevention benefits are derived as the ORV program reduces the number of potentially rabid raccoons, thereby reducing the human population at risk to *HPR*_*ORV*_ which ultimately reduces the number of PEPs and ATs.

Individuals’ perceived risk of rabies exposure may not decrease for several years after the actual prevalence of rabies declines [10], meaning people will continue to seek PEP even when treatment is unnecessary. Given this time lag, we assumed that *HPR*_*ORV*_ is not driven down for five years after the initiation of the program; therefore, no benefits accrue during this period. We further assumed that *HPR*_*ORV*_ declines at a linear rate, reaching its minimum in ten years and that without the program it is expected that the rabies epizootic would have continued indefinitely. Although the true length of the time lag is uncertain, the brevity of the epizootic supports the use of a relatively brief time lag which was further extended by allowing it to diminish gradually. Therefore, PEP and AT rates are assumed to remain elevated for 15 years after the implementation of the ORV program, and 10 years after the epizootic ended.

The difference between *HPR*_*baseline*_ and *HPR*_*ORV*_ represents the number of people no longer at risk (*HPR*_*saved*_) of rabies exposure due to the ORV program. *TB* of the program in a specific year was calculated as

$$TB_{t}=PEP_{saved,t}+AT_{saved,t}=\frac{HPR_{saved,t}}{100,000}[(PEP_{est}\cdot PEP_{cost}\text{)+(}AT_{est}\cdot AT_{cost}\text{)]}$$ (1)

where *PEP*_*est*_ and *AT*_*est*_ are estimates of the average rate of incidence of PEP and AT respectively per 100,000 people based on documented previous rabies epizootics in New Jersey [11], New York [12], and New Brunswick [13], and adjusted for pre-epizootic rates on Long Island. These estimates were used to determine the hypothetical case frequency that could have existed in the absence of a raccoon rabies ORV program [13]. New Jersey, New York and New Brunswick raccoon rabies epizootic PEP rates were reported as 66, 43.5, and 14; AT rates were reported as 483, 65, and 45 per 100,000 people, respectively. *PEP*_*cost*_ represents the total cost of rabies exposure per case, including direct and indirect costs, and was $4,203 [4]. Direct costs of PEP include vaccine costs and health professional salaries. Indirect costs include lost wages from missed work, travel time to receive treatment, expenditures on over-the-counter medications, and animal control measures. *AT*_*cost*_ reflects the costs associated with capture and testing of a suspected rabid animal and was estimated as $483 [4]. All dollar amounts are in 2008 U.S. dollars (see Table 2).

**Table 2:** Values and sources of variables in Eq 1.

Variable	Description	Value	Source
*HPR*	Human Population at Risk; comprised of the populations of Nassau and Suffolk Counties, 2007	2,864,793	US Census Bureau
*PEP*_*cost*_	Direct and indirect costs of receiving PEP	$4,203	Shwiff et al., 2007 [4]
*AT*_*cost*_	Cost of animal testing	$483	Shwiff et al., 2007 [4]
*PEP*_*est*_	Number of PEPs per 100,000 people avoided due to ORV program; average of reported rates from epizootics in New Jersey, New York, and New Brunswick and adjusted for pre-epizootic rates on Long Island	38.33	Uhaa et al., 1992 [11]; Wyatt et al., 1999 [12]; Shwiff et al., 2013 [13]
*AT*_*est*_	Number of ATs per 100,000 people avoided due to ORV program; average of reported rates from epizootics in New Jersey, New York, and New Brunswick and adjusted for pre-epizootic rates on Long Island	170.12	Uhaa et al., 1992 [11]; Wyatt et al., 1999 [12]; Shwiff et al., 2013 [13]

Total costs (*TC*) of the ORV program were calculated as the sum of bait and distribution costs. The program was funded by New York State and Nassau and Suffolk Counties. Actual costs were available for baits and a portion of the air distribution. However, the remaining air distribution and all of the ground distribution costs were provided by in-kind donations to the program and were not tracked. As a proxy, we used estimates of ground and air distribution costs of $20.43/km^2^ and $28.09/km^2^, respectively [14]. It should be noted that some costs may not be captured in this analysis. For example, costs of awareness campaigns, labor associated with organization and implementation of the program, etc. are not directly measured but should still be considered.

Program efficiency can be measured in two ways: benefit-cost ratios (BCRs) and net benefits. BCRs were calculated as total benefits divided by total costs, providing an indication of the returns for every dollar spent. Thus, a BCR greater than one indicates an efficient use of resources because the program’s benefits outweigh the costs. Similar information is provided by the measurement of net benefits, which is simply the total benefit minus the total cost.

A sensitivity analysis was performed to evaluate the effect of a range of PEP and AT rate estimates on the total benefits of the program. Rates were allowed to vary by as much as 70% and resulting benefits and BCRs were calculated.

### Macroeconomic Impacts

Reducing human exposure to raccoon rabies can produce regional macroeconomic impacts including changes in income and employment that arise from multiple sources. Macroeconomic impacts illustrate how reducing the prevalence of rabies affects people who were not at direct risk of the disease. Macroeconomic models, such as REMI PI+ (Regional Economic Models, Inc.), allow the estimation of impacts in terms such as income and employment, which are important to the general public. We estimated macroeconomic impacts arising from two sources: less income lost due to fewer people receiving PEP and shifts from healthcare spending to spending in other sectors of the economy.

The loss of income while seeking PEP reduces consumer spending throughout other sectors of the economy and leads to income and employment declines in those sectors. Thus, when the prevalence of the disease is reduced this harmful impact is lessened and regional income and employment will rise. Positive macroeconomic effects associated with a decrease in spending on PEP arise because a large portion of spending on PEP immediately leaves the state (rabies vaccines and human rabies immune globulin (HRIG) are produced outside New York State). Based on prices obtained from rabies vaccine and HRIG manufacturers, it was estimated that 84% of the direct costs of PEP are for vaccine/HRIG and the remaining 16% are for other medical costs (physician’s salary, etc.). Therefore, when PEP spending is reduced there will be a significant shift in spending to in-state businesses, ultimately resulting in an increase in income and employment in those industries. However, this positive impact is partially offset by a decrease in spending in the healthcare sector in New York, equal to the non-vaccine/HRIG portion of PEP costs.

The REMI model that was used to estimate the impact of the ORV program on New York State’s economy was a 70-sector REMI PI+ model of the New York economy. REMI is a computer-based simulation model of the US economy that allows modeling at both the national and sub-national scales. This structural economic forecasting model uses a non-survey based input-output (I-O) table like other widely-used, ready-made models, but links its I-O table to thousands of simultaneous equations to overcome the rigidness of static I-O models [15]. By incorporating the strengths of input-output, computable general equilibrium, and econometric methodologies, REMI is able to overcome the limitations of using a single methodology. This dynamic forecasting and policy tool has the ability to generate annual forecasts and simulations that detail behavioral responses to compensation, price, and other economic factors. The structure of the model incorporates inter-industry transactions, endogenous final demand feedbacks, substitution among factors of production in response to changes in expected income, wage responses to changes in labor market conditions, and changes in the share of local and export markets in response to the change in regional profitability and production costs. The model is constructed based on national, state, and county level data from the Bureau of Economic Analysis, Bureau of Labor Statistics, and the Bureau of the Census, as well as forecasts from the Research Seminar in Quantitative Economics at Michigan State University.

## Results

Total benefits resulting from the ORV program were the savings from avoided PEPs and ATs over the course of the program (2005–10) and projected nine years into the future (Table 3). The first five years of the program show no benefits due to the assumed time lag between initiation of the program and the reduction of the human population at risk. Benefits then increase linearly over the next ten years ultimately reaching the maximum, pre-epizootic level. Costs of the ORV program totaled approximately $2.6 million (Table 4). Broad-scale rabies intervention ended in 2010; accordingly, costs for subsequent years are zero.

**Table 3:** Benefits and BCRs of the Long Island ORV program in 2008 dollars.

Year	PEP Avoided	PEP Savings	AT Avoided	AT Savings	Total/year	Discounted 3%	Cumulative	BCR
2010	110	$461,481	487	$235,390	$696,870	$583,618	$583,618	0.22
2011	220	$922,962	975	$470,779	$1,393,741	$1,133,239	$1,716,857	0.43
2012	329	$1,384,443	1,462	$706,169	$2,090,611	$1,650,348	$3,367,205	0.63
2013	439	$1,845,923	1,949	$941,558	$2,787,482	$2,136,373	$5,503,577	0.82
2014	549	$2,307,404	2,437	$1,176,948	$3,484,352	$2,592,685	$8,096,263	0.99
2015	659	$2,768,885	2,924	$1,412,338	$4,181,223	$3,020,604	$11,116,867	1.15
2016	769	$3,230,366	3,411	$1,647,727	$4,878,093	$3,421,396	$14,538,263	1.31
2017	878	$3,691,847	3,899	$1,883,117	$5,574,963	$3,796,279	$18,334,542	1.45
2018	988	$4,153,328	4,386	$2,118,506	$6,271,834	$4,146,421	$22,480,963	1.58
2019	1,098	$4,614,809	4,873	$2,353,896	$6,968,704	$4,472,946	$26,953,909	1.71

**Table 4:** Costs of the Long Island ORV program in 2008 dollars.

Year	Baits	Distribution	Total/year	Discounted 3%	Cumulative
2005	$197,216	$19,446	$216,662	$210,351	$210,351
2006	$465,670	$30,574	$496,244	$467,757	$678,108
2007	$536,384	$60,579	$596,963	$546,306	$1,224,414
2008	$560,006	$116,768	$676,774	$601,305	$1,825,719
2009	$495,040	$29,187	$524,227	$452,203	$2,277,922
2010	$382,200	$22,310	$404,510	$338,770	$2,616,692

Net benefits are the total benefits minus the total costs. As of 2016, cumulative benefits for New York exceeded $14 million (Table 3). Cumulative benefits through 2019 are projected to reach almost $27 million while cumulative costs were approximately $2.6 million (Table 4), leading to a projected net benefit of the ORV program through 2019 exceeding $24 million. Comparing the benefits with the costs of the program for each year gives the benefit-cost ratios (BCRs). The cumulative BCR (benefit each year divided by the total cost) is projected to reach 1.71 in 2019, indicating every dollar spent on the ORV program will save $1.71 in costs.

Sensitivity analysis revealed positive benefits and BCRs greater than one (Table 5), even when estimated PEP and AT rates were reduced by 40% (to 23.00 and 102.07, respectively), revealing that the benefits of the ORV program are robust to broad changes in model parameters. However, if PEP rates drop to levels seen in a previous epizootic in New York (12.1) [2], then this ORV program would no longer be economically efficient (BCR < 1).

**Table 5:** Total benefits and BCRs resulting from adjusting estimated PEP and AT rates.

	# PEP	#AT	BCR	Total Benefits
Baseline	38.33	170.12	1.71	$26,953,909
70% fewer PEP and AT	11.50	51.04	0.51	$8,086,173
40% fewer PEP and AT	23.00	102.07	1.03	$16,173,620
20% fewer PEP and AT	30.66	136.09	1.37	$21,563,127
20% more PEP and AT	45.99	204.14	2.05	$32,344,691
40% more PEP and AT	53.66	238.16	2.39	$37,734,653
70% more PEP and AT	65.16	289.20	2.91	$45,821,646

Regional economic modeling predicted employment growth of 106 jobs and a Gross Domestic Product (GDP) increase of $9.2 million through 2019 due to increased consumer spending resulting from avoided PEPs and ATs (Table 6). This takes into account the spending offset in the medical sector by including only saved costs that would have gone out of state (vaccines and HRIG) and the avoided costs of lost wages, for which there is no offset.

**Table 6:** REMI results indicating the macroeconomic impacts of the ORV program to New York State (GDP in thousands of dollars).

	2010	2011	2012	2013	2014	2015	2016	2017	2018	2019	Total
**Jobs**	4	5	6	8	12	12	12	12	18	17	106
**GDP**	128	384	512	640	1,024	896	1,408	1,024	1,536	1,664	9,216

## Discussion

This study provided a retrospective examination of the benefits and costs of a wildlife rabies elimination program on Long Island that incorporated, for the first time, the macroeconomic impacts of reduced rabies burden to the New York State economy. Other studies examining the benefits and costs of successful elimination of rabies from an area have been conducted but have never estimated the broader macroeconomic implications [7, 13]. The domestic dog/coyote variant of rabies was eliminated from Texas by moving a zone of immunity south to the Mexican border and maintaining the zone at the U.S.-Mexico border to prevent reintroduction. In Quebec, a variety of techniques including ORV and point-infection control were used to successfully eliminate an outbreak of raccoon rabies. While both of these studies provided a benefit-cost analysis of the elimination program, an examination of impacts to those not directly impacted by rabies exposure was absent.

Regional economic modeling was used to estimate the macroeconomic effects of the ORV program on the New York economy in terms of employment and GDP (income). As the ORV program protected individuals in Suffolk and Nassau counties, fewer individuals had to receive PEP. Avoided expenditures on PEP were reflected as increased consumer spending. While some of the gain to individuals was offset by a loss to the medical sector, the REMI model predicted positive impacts on jobs and income in New York as a result of the program. Estimating the impact on employment and state GDP of controlling a primarily wildlife-based disease is largely absent in the literature. This is the first estimation of the broader implication of rabies to the macroeconomy. This type of analysis is important because it links the impacts of the disease control (ORV) program to individuals who were otherwise not involved in the program through tangible concepts such as changes in employment and income. These individuals may have been aware that a program was being conducted in their area, but may not have been able to discern any personal benefit.

A challenge of this study was determining the hypothetical annual frequencies of public health interventions (PEP and AT) that would have existed in the absence of a raccoon rabies control program. These data were needed to calculate damages avoided in the economic analysis. Because rabies control tactics were initiated within days of identification of the first terrestrial case in 2004, the estimated frequencies were based on information from previous raccoon rabies epizootics occurring in New Brunswick, New Jersey and New York. The use of these average and adjusted frequencies reduces uncertainty of the monetary value of damages avoided. In addition, a sensitivity analysis revealed that large changes in estimated PEP and AT rates would still result in BCRs greater than one. The effect of public perception of risk on PEP rates is not fully understood. However, we do know that a decrease in rabies prevalence does not result in an immediate decrease in the number of PEP sought, indicating that public perception of rabies risk is not acutely responsive to actual rabies risk. Consequently, minor changes in actual rabies prevalence (e.g., due to cyclical disease dynamics within a wildlife population) are not likely to have a significant effect on PEP and AT rates.

The potential for rabies to advance into pre-epizootic areas that are located beyond existing ORV-barrier zones, as well as viral emergence within previously-vaccinated areas where the virus appears to be extirpated, will continue through long distance raccoon dispersals, as well as human-assisted animal translocations that are purposeful or accidental. A rabid juvenile male raccoon was dispatched and submitted by a police officer in Nassau County on 23 March 2016, with subsequent raccoon variant determination at the NY State Health Department Rabies Laboratory on 24 March. The animal was located approximately 750m from the southern-most rabid raccoon that was previously reported 11 years earlier during October 2005. The most recent historical case of terrestrial rabies in Nassau County was confirmed on 2 November 2007. The first phases of a contingency response plan were effected immediately following detection of the March 2016 case. Local cooperators (e.g., police, park personnel, animal control officers) were notified and enhanced surveillance for sick and abnormally-acting raccoons was increased in Nassau and Suffolk Counties. Resources were also in place for the planning and execution of an ORV campaign. Only the single, juvenile raccoon was confirmed rabid during subsequent spring and summer months. Accordingly, discussions with county and state agency personnel resulted in a decision to delay the proposed ORV campaign indefinitely, pending detection of additional viral activity. In comparison, enhanced rabies surveillance efforts during the initial 2004 epizootic yielded the confirmation of seven rabid raccoons during the first 35 days. ORV managers will continue to react and make informed decisions on a case-by-case basis, until the raccoon rabies variant is eradicated from the extent of its range in North America.

A comprehensive picture of the economic impacts resulting from elimination of wildlife diseases is crucial to understanding the benefits of control programs. Vaccination of wildlife, companion animals, and livestock against rabies is critical for elimination of the disease. The next step is choosing the most efficient and effective method available. This analysis suggests that baiting to eliminate rabies in a geographically constrained area can provide positive economic returns despite a five-year temporal delay before financial benefits start to accrue. The real and perceived risks associated with rabies exposure and PEP in small intervention areas must ultimately be compared to large-scale vaccination programs that are designed with the goal of regional rabies elimination within extensive land areas. Financial benefits to society may be negatively impacted if public and professional anxieties remain at a heightened level in narrow disease elimination zones where the threat of rabies exposure persists in close proximity. The estimated macroeconomic impacts over time demonstrate the societal value of rabies elimination in a wildlife reservoir species. Such programs are often perceived as too costly or lacking a measurable public health impact. Quantitative analyses as performed in this study are important for public health policy makers at both local and national levels. The results from this study can be used in future ORV and rabies elimination planning.
